# Primary pulmonary primitive neuroectodermal tumor metastasis to the pancreas: a rare case with seven-year follow-up

**DOI:** 10.1186/1746-1596-8-51

**Published:** 2013-03-27

**Authors:** Lin Shi, Zhanlin Guo, Xinlin Wu

**Affiliations:** 1 Department of Pathology, The First Affiliated Hospital of Inner Mongolia Medical University, Huhhot, 010059, China; 2 Institute of pathology and pathophysiology, Inner Mongolia Medical University, Huhhot, 010059, China; 3 Department of Cardiovascular and Thoracic Surgery, The First Affiliated Hospital of Inner Mongolia Medical University, Huhhot, Inner Mongolia, 010059, P. R. China; 4 Department of General Surgery, The First Affiliated Hospital of Inner Mongolia Medical University, Huhhot, 010059, China

**Keywords:** Primitive neuroectodermal tumor, Lung, Metastasis, Pancreas

## Abstract

**Abstract:**

There are only nine primitive neuroectodermal tumor (PNET) cases that have arisen in lung parenchyma without pleural or chest wall involvement in the literature. Here, we present a long–term survival case of pulmonary PNET. A pulmonary mass was detected in a 19-year-old man on a chest radiograph and computed tomography image. At the three-year follow-up, the mass had enlarged in diameter by two-fold. The lesion was resected via lower left lobectomy. Histologically, the tumor was composed of uniform cells with round nuclei and scanty cytoplasm arranged in lobules with rosettes and pseudorosettes formation. Immunohistochemically, the tumor was positive for CD99, vimentin, neuron specific enolase and chromogranin A, and negative for cytokeratins, CD3, desmin, and leukocyte common antigen. Pancreatic metastasis occurred sixteen months after the first surgery, which was managed by pancreatectomy. The patient has survived seven years after the mass was initially detected, and four years after the first lobectomy.

**Virtual slides:**

The virtual slide(s) for this article can be found here: http://www.diagnosticpathology.diagnomx.eu/vs/1500847644913244

## Background

Primitive neuroectodermal tumors (PNETs), first described by Hart and Earle in 1973 [1], belong to the group of highly malignant neoplasms and have a tendency toward early metastasis. This rare neoplasm is more prevalent in children and adolescents than in adults, as well as in the central than the peripheral nervous system. Peripheral PNETs (pPNETs) usually involve bone or soft tissues but have also been discovered in a variety of other organs, such as the kidney, urinary bladder, and heart [2-4]. Reports of pPNETs that arise in the lung parenchyma without pleural or chest wall involvement are extremely rare. Herein, we report an additional case of primary PNET in the lung, which then metastasized to the pancreas.

## Case presentation

**Figure 1:**
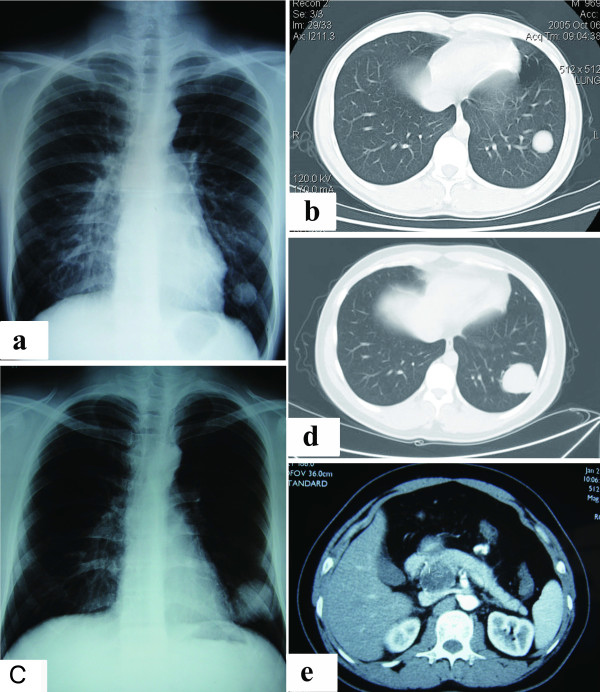
**Chest radiographs and CT scans of lung and pancreas. a**) Chest radiograph showing a mass in the left lung (October 2005). **b**) Chest CT showing a well-defined solid mass in the lower lobe of the left lung (October 2005). **c)** Chest radiograph showing an enlarged mass in the left lung (September 2008). **d**) Chest CT showing an enlarged mass in the lower lobe of the left lung (September 2008). **e**) Contrast-enhanced CT showing a huge solid mass with cystic areas originating from the pancreas.

In October 2005, a mass in the left lower lung lobe of a 19-year-old male was detected on a chest radiograph and a computed tomography (CT) image during a routine health examination (Figure 1a, b). Because the patient was young and asymptomatic, and since the CT image indicated a benign tumor, a lung biopsy was not performed at the time. Physicians then recommended the patient for semi-annual follow-up.

At the follow-up two-and-a-half years later, an imaging study showed the invariable lung tumor. At the year 3 follow-up, the mass was found to have enlarged remarkably. The patient was admitted to our hospital in September 2008 for further evaluation of the lung mass. A chest radiograph and CT scan showed a well-circumscribed tumor with a diameter of 5.0 cm in the lateral basal segment of the left lung (Figure 1c, d), but did not detect any bilateral hilar lymphadenopathy. A bronchoscopy showed no abnormal results. Standard staging procedures, including brain magnetic resonance imaging (MRI), abdominal ultrasonography, and bone scintigraphy, did not detect distant metastasis.

**Figure 2:**
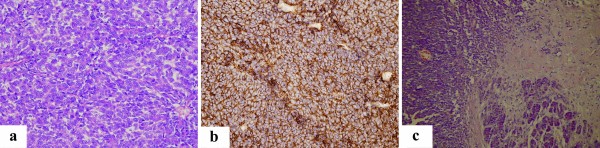
**Histologic features of the tumor in the lung and pancreas. a**) The pulmonary tumor cells had round or oval nuclei, a fine chromatin pattern, and indistinct cytoplasm and nucleoli. Mitotic activity was very high (HE, 400×). **b**) The pulmonary tumor cells showed a consistent and strong membranous expression of CD99 (SP, 400×). **c**) Diffusely infiltrative tumor cells were present in the pancreas parenchyma (HE, 200×).

A lobectomy of the lower left lung combined with a lymph node dissection was ordered. During the operation, the surgeon discovered a well-defined 5.5 cm × 5.0 cm solid mass in the parenchyma of the lower lobe of the left lung, without pleural or chest wall involvement. Microscopic analysis of the resected tissue revealed that the tumor was composed of small, round cells with inconspicuous cytoplasm and arranged in diffuse or compact sheets or lobules (Figure 2a). Immunohistochemical staining showed that the tumor cells were strongly positive for vimentin and CD99 (Figure 2b), focally positive for neuron specific enolase (NSE) and chromogranin A (CgA), and negative for cytokeratins, CD3, desmin, and leukocyte common antigen (LCA). Immunophenotype analysis ruled out lymphoma, desmoplastic small round cell tumor, neuroblastoma, small-cell carcinoma, rhabdomyosarcoma, and monophasic synovial sarcoma. Therefore, the postoperative pathologic diagnosis was PNET of the lung.

The subsequent pathology indicated clear surgical margins and pleura and lymph nodes free of tumor cells. The postoperative course was uneventful. The patient refused adjunct radiation or chemotherapy after the initial surgery, but participated in regular follow-up examinations.

After a 16-month disease-free period, the patient complained of several episodes of a dull ache in the upper right quadrant of the abdomen. The patient was admitted to our hospital for further evaluation in February 2010. An ultrasonography and contrast-enhanced CT scan of the abdomen revealed a 3.5 cm × 4.0 cm mass in the pancreatic head (Figure 1e). The mass had solid and cystic characteristics and was difficult to distinguish from the surrounding pancreas parenchyma. Laparotomy showed a 3.0 cm × 4.0 cm solid and cystic mass on the surface of the uncinate process of the pancreas. The boundary between the mass and pancreas parenchyma was obscure and the mass appeared unencapsulated. An *en bloc* resection of the mass was accomplished through the standard Whipple procedure [5]. Diffuse infiltrative tumor cells were present in the surrounding tissue (Figure 2c). Immunohistochemical staining of the tumor cells confirmed strong membranous expression of CD99 and focal expressions of vimentin and synaptophysin.

The immunophenotyping results and patient history of pulmonary PNET indicated a postoperative pathologic diagnosis of metastatic pancreatic PNET. Again, the patient refused adjunct radiation and chemotherapy. To date, no evidence of tumor recurrence or metastasis has been found. The patient has survived seven years after the mass was initially detected, and four years after the first radical resection.

## Discussion

pPNETs belong to the family of “small round cell tumors” that show varying degrees of neuroectodermal differentiation and are derived from cells originating from the neural crest [6] and are characterized by a specific chromosomal translocation, t(11;22)(q24;q12), in most cases. Among the reported cases of PNET, tumors involving the thoracopulmonary region were first reported as “malignant small cell tumors of the thoracopulmonary region in childhood” by Askin et al. in 1979, which led to these tumors being referred to as Askin’s tumors [7].

Conventional light microscopy analysis of PNETs shows undifferentiated small, round cells with uniform, unconspicuous nuclei and scanty cytoplasm arranged in lobules with rosettes and pseudorosettes formation; in addition, there is little or no stroma. Immunohistochemically, PNETs are positive for CD99 antigen, but CD99 immunostaining is not specific and the results must be interpreted in combination with other findings. T lymphoblastic lymphoma, poorly differentiated synovial sarcomas, stromal tumors, and rare rhabdomyosarcoma may show CD99 positivity. Vimentin stains most tumor cells and neural markers, such as NSE, and is frequently expressed by tumor cells [6]. Cytokeratin-positive staining has also been reported in some cases of primitive neuroectodermal tumors [6]. To diagnose a tumor as PNET, it should display small round cells forming rosette and pseudorosettes, and should be positive for at least two of the neural markers. Ultrastructural analysis usually shows PNET cells to have complex cytoplasmic processes, microtubules, and few neurosecretory granules. The following chromosomal translocations have been associated with PNET specimens: t(21;22)(q22;q12), t(11;22)(q24;q12), t(7;22)(p22;q12), and t(7;22)(q22;q12) [8]. Thus, the diagnosis of PNET necessitates histopathological, immunohistochemical, ultrastructural, and, if possible, genetic analyses.

The differential diagnosis of PNETs includes neuroblastoma, lymphoma, small-cell carcinoma, rhabdomyosarcoma, monophasic synovial sarcoma, and desmoplastic small round cell tumor, all of which are indistinguishable by conventional light microscopy [6]. However, due to the different prognostic characteristics and therapeutic schedules for each of these tumor types, differential diagnoses are essential. Immunohistochemical positivity for CD99, NSE, synaptophysine, and chromogranine A are very useful in differential diagnosis. Furthermore, the presence of Homer-Wright rosettes are typical for neuroblastomas, which are also positive for NSE, synaptophysine, and chromogranine A, but negative for CD99. LCA positivity supports the diagnosis of lymphoma, but T cell lymphoblastic lymphoma may be negative for LCA and positive for CD99 and CD3. Small-cell carcinoma is almost always positive for cytokeratin, while rhabdomyosarcoma is positive for desmin, actin, myoglobulin, and monophasic synovial sarcoma is positive for CD99, cytokeratin , EMA. The desmoplastic small round cell tumor is characterized by sharply circumscribed nests or clusters of small, undifferentiated cells surrounded by a desmoplastic stroma, and show positivity for cytokeratin and desmin, but negativity for CD99. Therefore, the phenotypes observed in our case, i.e., positive expression of CD99, vimentin, NSE, and synaptophysin, and negative expression of cytokeratins, CD3, desmin, and LCA, are highly suggestive of a pulmonary PNET [3,9-11].

Despite the patient’s history of pulmonary PNET and imaging findings that were consistent with primary pancreatic cancer, we initially suspected primary serous cystic pancreatic neoplasm because metastasis of a PNET to the pancreas had never been previously reported. However, the patient’s histological findings indicated no transition had occurred from the pancreas to the neoplastic tissue. In addition, there were no clinical signs of chronic pancreatitis in the surrounding parenchyma. Considering the expression of CD99, NSE, and synaptophysine, as well as the primary pulmonary PNET, we believed the findings strongly supported a diagnosis of metastatic pancreatic PNET.

According to the literature, there have only been nine reported cases of PNET originating from the lung without pleural or chest wall involvement [9,12-17]. Ages of the reported patients have ranged between 8 to 67 years, with a mean age of 33 years. There is a slight male predominance, with the male:female ratio being 5:4. Five of the cases originated from the left lung and four from the right. Despite treatment with various combinations of surgery, chemotherapy, and radiation therapy, the survival rate in the previous reports was poor; typically, the two-year survival rate after operation is 33.3%. Of the nine reported cases, seven were followed-up; four were alive without disease at eight months, 16 months, 22 months and two years after surgery, and three had died at three months, two years, and two years after operation due to local recurrence or widespread metastatic disease. Furthermore, the predominating metastatic sites are the lungs, adrenal glands, and ovaries. In our case, no adjunct radiation or chemotherapy was given after the initial surgery, due to patient refusal. However, recurrence was detected at sixteen months after the first resection and appeared as a metastasis to the pancreas. The patient underwent the second radical surgical resection of the metastatic tumor. Adjunct radiation or chemotherapy again refused by the patient after the second surgery. To date, no evidence of tumor recurrence or metastasis has emerged. The patient has survived 32 months after the second surgery. The long-term survival of the patient may have been a result of the two radical surgeries alone, but we believe that his prognosis would have been better if the lung biopsy was performed promptly in October 2005. We speculate that close follow-up with immediate surgical intervention when required may have helped to prolong the survival of our case [18].

## Conclusion

We reported a rare case in which a primary pulmonary primitive neuroectodermal tumor metastasized to the pancreas. The primary and metastatic tumors were resected respectively, and no adjunct radiation or chemotherapy was given before or after the two surgical procedures. This newly described PNET patient has the longest survival (seven years) and the longest post-operational follow-up period (four years) of all the cases reported in the literature to date. We speculate that close follow-up with immediate surgical intervention when required may have helped to prolong the survival of our case.

## Consent

Written informed consent was obtained from the patient for publication of this case report and accompanying images. A copy of the written consent is available for review by the Editor-in-Chief of this journal.

## Competing interests

The authors declare that they have no conflict of interest.

## Authors’ contributions

SL gave the final histopathological diagnosis, performed the literature review, acquired photomicrographs, and drafted the manuscript. GZL participated in patient management and edited and revised the manuscript. WXL participated in patient management and drafted the manuscript. All authors read and approved the final manuscript.

## References

[B1] Hart MN Earle KM Primitive neuroectodermal tumors of the brain in children Cancer 1973 32 890 897 10.1002/1097-0142(197310)32:4<890::AID-CNCR2820320421>3.0.CO;2-O 4751919

[B2] Takeuchi T Iwasaki H Ohjimi Y Kaneko Y Ishiguro M Fujita C Miura Y Hiratsuka Y Sakamoto K Kikuchi M Renal primitive neuroectodermal tumor: an immunohistochemical and cytogenetic analysis Pathol Int 1996 46 292 297 10.1111/j.1440-1827.1996.tb03613.x 8726854

[B3] Mentzel T Flaschka J Mentzel HJ Eschholz G Katenkamp D Primary primitive neuroectodermal tumor of the urinary bladder: clinicopathologic case report and differential small cell tumor diagnosis of this site Pathologe 1998 19 154 158 10.1007/s002920050269 9556802

[B4] Charney DA Charney JM Ghali VS Teplitz C Primitive neuroectodermal tumor of the myocardium: a case report, review of the literature, immunohistochemical, and ultrastructural study Hum Pathol 1996 27 1365 1369 10.1016/S0046-8177(96)90352-4 8958313

[B5] Tran KT Smeenk HG Van Eijck CH Kazemier G Hop WC Greve JW Terpstra OT Zijlstra JA Klinkert P Jeekel H Pylorus preserving pancreaticoduodenectomy versus standard Whipple procedure: A prospective, randomized, multicenter analysis of 170 patients with pancreatic and periampullary tumors Ann Surg 2004 240 738 745 10.1097/01.sla.0000143248.71964.29 15492552 PMC1356476

[B6] Christopher DM Fletcher K Krishnan U Fredrik M World Health Organization classification of tumours Pathology and genetics of tumours of soft tissue and bone 2002 2 Lyon: IARC Press 299

[B7] Askin FB Rosai J Sibley RK Dehner LP McAlister WH Malignant small cell tumor of the thoracopulmonary region in childhood: a distinctive clinicopathologic entity of uncertain histogenesis Cancer 1979 43 2438 2451 10.1002/1097-0142(197906)43:6<2438::AID-CNCR2820430640>3.0.CO;2-9 222426

[B8] Kuroda M Urano M Abe M Mizoguchi Y Horibe Y Murakami M Tashiro K Kasahara M Primary primitive neuroectodermal tumor of the kidney Pathol Int 2000 50 967 972 10.1046/j.1440-1827.2000.01147.x 11123763

[B9] Mikami Y Nakajima M Primary pulmonary primitive neuroectodermal tumor (PNET): a case report Pathol Res Pract 2001 97 113 119 discussion 121–122 11261815 10.1078/0344-0338-00019

[B10] Cetiner H Kir G Gelmann EP Ozdemirli M Primary vulvar Ewing sarcoma/primitive neuroectodermal tumor: a report of 2 cases and review of the literature Int J Gynecol Cancer 2009 19 1131 1136 10.1111/IGC.0b013e3181acae33 19820381

[B11] Colecchia M Dagrada G Poliani PL Messina A Pilotti S Primary primitive peripheral neuroectodermal tumor of the prostate: immunophenotypic and molecular study of a case Arch Pathol Lab Med 2003 127 e190 e193 12683899 10.5858/2003-127-e190-PPPNTO

[B12] Imamura F Funakoshi T Nakamura S Mano M Kodama K Horai T Primary primitive neuroectodermal tumor of the lung: report of two cases Lung Cancer 2000 27 55 60 10.1016/S0169-5002(99)00078-1 10672784

[B13] Tane S Nishio W Hashimoto S Hokka D Maniwa Y Funada Y Kotani Y Hirai C Ohbayashi C Yoshimura M Ewing’s sarcoma family of tumors originating in the main bronchus Thoracic Cancer 2012 3 353 356 10.1111/j.1759-7714.2011.00105.x 28920284

[B14] Paik SH Park JS Koh ES Kim HK Shin HK Hong HS Cha JK Lee HK Primary pulmonary primitive neuroectodermal tumor: CT and skeletal scintigraphic image features with pathologic correlation Eur Radio 2006 16 2128 2131 10.1007/s00330-006-0216-3 16865370

[B15] Takahashi D Nagayama J Nagatoshi Y Inagaki J Nishiyama K Yokoyama R Moriyasu Y Okada K Okamura J Primary Ewing’s sarcoma family tumors of the lung – a case report and review of the literature Jpn J Clin Oncol 2007 37 874 877 10.1093/jjco/hym108 17998263

[B16] Lee YY Kim Do H Lee JH Choi JS In KH Oh YW Cho KH Roh YK Primary pulmonary Ewing’s sarcoma/primitive neuroectodermal tumor in a 67-year-old man J Korean Med Sci 2007 22 Suppl S159 S163 10.3346/jkms.2007.22.S.S159 17923745 PMC2694395

[B17] Tsuji S Hisaoka M Morimitsu Y Hashimoto H Jimi A Watanabe J Eguchi H Kaneko Y Peripheral primitive neuroectodermal tumour of the lung: report of two cases Histopathology 1998 33 369 374 10.1046/j.1365-2559.1998.00485.x 9822928

[B18] Ouarssani A Atoini F Lhou FA Idrissi MR Desmoplastic small round cell tumor of the pleura Thoracic Cancer 2011 2 117 119 10.1111/j.1759-7714.2011.00046.x 27755831

